# N170 ERPs could represent a logographic processing strategy in visual word recognition

**DOI:** 10.1186/1744-9081-3-21

**Published:** 2007-04-23

**Authors:** Gregory Simon, Laurent Petit, Christian Bernard, Mohamed Rebaï

**Affiliations:** 1 Groupe d'Imagerie Neurofonctionnelle, UMR 6194, CNRS CEA, Universities of Caen & Paris Descartes, GIP Cyceron, boulevard Henri Becquerel, 14074 Caen Cedex, France; 2 Laboratoire Psychologie et Neurosciences de la Cognition (EA1780), University of Rouen, rue Lavoisier, 76821 Mont Saint Aignan Cedex, France

## Abstract

**Background:**

Occipito-temporal N170 component represents the first step where face, object and word processing are discriminated along the ventral stream of the brain. N170 leftward asymmetry observed during reading has been often associated to prelexical orthographic visual word form activation. However, some studies reported a lexical frequency effect for this component particularly during word repetition that appears in contradiction with this prelexical orthographic step. Here, we tested the hypothesis that under word repetition condition, discrimination between words would be operated on visual rather than orthographic basis. In this case, N170 activity may correspond to a logographic processing where a word is processed as a whole.

**Methods:**

To test such an assumption, frequent words, infrequent words and pseudowords were presented to the subjects that had to complete a visual lexical decision task. Different repetition conditions were defined 1 – weak repetition, 2 – massive repetition and 3 – massive repetition with font alternation. This last condition was designed to change visual word shape during repetition and therefore to interfere with a possible visual strategy during word recognition.

**Results:**

Behavioral data showed an important frequency effect for the weak repetition condition, a lower but significant frequency effect for massive repetition, and no frequency effect for the changing font repetition. Moreover alternating font repetitions slowed subject's responses in comparison to "simple" massive repetition.

ERPs results evidenced larger N170 amplitude in the left hemisphere for frequent than both infrequent words and pseudowords during massive repetition. Moreover, when words were repeated with different fonts this N170 effect was not present, suggesting a visual locus for such a N170 frequency effect.

**Conclusion:**

N170 represents an important step in visual word recognition, consisting probably in a prelexical orthographic processing. But during the reading of very frequent words or after a massive repetition of a word, it could represent a more holistic process where words are processed as a global visual pattern.

## Background

Event-related potential N170 is a negative occipito-temporal component peaking around 170 ms after presentation of visual stimulus such as face, object or word. Despite its early latency, recent advances suggest that this component represents an important stage of processing in recognition of a visual pattern. The first well-established proofs were provided by comparing recognition processes involved in faces and objects. Numerous studies demonstrated larger N170 amplitudes for face than object and evidenced that only face's rotation affected N170 amplitude and latency [1-3]. Those differences were interpreted as the existence of a face-specific encoding, but recent works evidenced that N170 inversion effect can also occur during the processing of Greebles (a class of fictional objects) when subjects were trained to recognize them [4]. This last result contradicted the face-specific processing hypothesis and raised the question of expertise for this component. But more important, Bentin and Golland [5] suggested that face N170 associated with extrastriate visual mechanisms is modulated by top-down processes, because the same stimuli can elicit different amplitudes as a function of subject's knowledge about these stimuli (see also Jemel and colleagues [6]).

The N170 component appears then as a main step in visual pattern extraction and recognition for face and object but the question of its specificity is still debated as well as its functional signification during word reading. Only one study compared directly N170 elicited by faces, objects and words. This component was right lateralized for faces, smaller and bilateral for objects such as cars, and strongly left lateralized for printed words [7]. According to Vigneau and colleagues, the hemispheric lateralization may be a better marker of the functional brain specialization than some increased activity at a given anatomical location [8] and the N170 component represents the earliest left lateralized component during word recognition. As a matter of fact, in the domain of visual word recognition, both ERP and MEG experimental data comparing the processing of orthographic stimuli with that of pseudoletter strings (e.g., false font, ASCII symbols,...) reported a greater N170 leftward negativity for orthographic stimuli [9-12]. Those modulations were interpreted as related to the expertise for letters or well-ordered letter strings and suggest the existence of an orthographic encoding stage for N170 in the left hemisphere. In addition, a combined fMRI and ERP study evidenced a positive correlation between the amplitude of the late part of N170 and the metabolic activity in the visual word form area (VWFA) [13]. This left fusiform area, as well as the N170 component, may be dedicated to prelexical letter processing because of similar activations usually reported for words of different semantic categories and pseudowords [8,9,14].

However in the literature, some studies reported a N170 word frequency effect that was often interpreted as a lexical access modulation. This frequency effect and this interpretation are clearly in disagreement with the hypothesis of a prelexical orthographic processing for N170 elicited by words. But in these studies, words were irregular/lexical ambiguous [15,16] or presented tachistoscopically [17], some parameters that may have an impact on orthographic encoding. Interestingly, MEG studies of Assadollahi and Pulvermuller [18,19] evidenced a word frequency effect close to the N170 latency, using repeated stimuli. Simon and colleagues [11] also obtained a N170 word frequency effect during a lexical decision task but only when a massive repetition was used. In this last experiment, repeated frequent words elicited a larger N170 amplitude than infrequent ones, the same effect as the face familiarity found during massive repetition by Caharel and colleagues [20,21]. We can thus suppose that repetition plays an important role in N170 word frequency effect.

As a matter of fact we decided in this study to investigate the interaction between the word lexical frequency and the repetition of stimuli on N170 component. Indeed the repetition can be used in experimental paradigm in order to "artificially" modulate the lexical frequency of words. One may argue that the N170 elicited by massively repeated words may represent a different processing that the ones elicited by words presented in "standard" conditions. But if we consider that the N170 is usually associated to orthographic encoding – at least for its early part – (i.e. letter recognition), what kind of processing favors repetition? The more coherent interpretation would be that under word repetition effect, discrimination between stimuli would be operated on visual rather than orthographic basis. In this case, such a processing may be assimilated to a logographic stage [22] in which words are not recognized as a string of letters but rather as a whole visual pattern (see also, [23]), applying holistic processes as in face perception. The MEG study of Tarkiainen and colleagues evidenced that even though the hemispheric balance was different around 150 ms for face and letter-string, the activated areas within the inferior occipito-temporal cortex were very close to each other [24].

In fact, the understanding of whether the visual word recognition is assumed only on the basis of letters or with other pertinent sources such as global visual pattern, is an important and recurrent question concerning alphabetic languages [25]. To test the effects of word perceptual familiarity and therefore indirectly the hypothesis of a logographic processing in adults, most paradigms created subtle but significant perceptive deformations of the stimuli in order to estimate their impact on the visual word recognition. As a matter of fact, the modification of the word's visual shape is supposed to disrupt the logographic processing. Numerous perceptual transformations have been used, such as contrast manipulation [26] or cAsE MixIng [25,27-30]. But these experiments produced heterogeneous behavioral results that are difficult to interpret. According to a fMRI study of Polk and Farah [31], no significant difference in activation was obtained comparing pure-case and alternating-case words in the left VWFA. This result suggests that the response of this area and therefore probably the N170 amplitude would not be modulated by perceptual familiarity. In this case, it appears difficult to explain the N170 word frequency effect evidenced in some studies.

We performed the present study in order to investigate the word frequency effect on N170 and to test the hypothesis of the emergence of a visual/logographic process during a massive repetition. EEG was recorded continuously as subjects performed a lexical decision task with three mixed repetition modalities. This task was chosen in order to ensure lexical access and therefore behavioral word frequency effects. The first repetition modality consisted in the presentation of lists of frequent words, infrequent words and pseudowords presented only twice (weak repetition). The second repetition condition consisted in 100 repetitions for each type of stimulus (massive repetitions). The third was the same that the previous but stimuli were repeated with different fonts (alternating font repetitions). The font manipulation was chosen instead of a case mixing because we estimated that it allows to disrupt the logographic processing without an important disturbance of word recognition when simple fonts are used. The hypothesis was that if alternating case fonts led to a disappearance of the N170 frequency effect despite of a massive repetition, it would attest for the use of a visual/logographic strategy in the word recognition. Moreover, in order to assess the evolution of the repetition effect through the time, we contrasted electrophysiological data obtained at the beginning of the experiment with the ones of the end of session.

## Methods

### Participants

Twenty-eight literate adults (14 men and 14 women), aged 20–30 years, with no history of neurological disease or learning impairment, participated to the behavioral study. Ten of them (5 men and 5 women) were then selected for ERPs. All of the subjects had normal or corrected-to-normal vision, were right-handed (Edinburgh test [32]), and had no previous history of neuropsychiatric disorders.

### Stimuli

**Figure 1:**
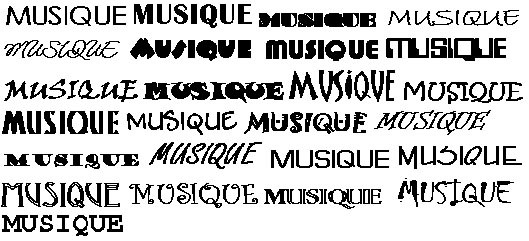
Fonts used for repetition with font alternation condition.

The stimuli consisted of frequent words, infrequent words and pseudowords presented according to three conditions: 1 – weak repetitions, 2 – massively repeated items and 3 – massively repeated items with different fonts. For the weak repetitions condition, lists of 50 frequent words, 50 infrequent words and 50 pseudowords were constituted. Lexical frequencies of the words were assessed with the Brulex French database [33] and were significantly different (p < .001) between the frequent and infrequent lists (mean: 11.054 and 160 per 100 million respectively). Moreover, in both lists, the words had an identical mean of 7 letters, were constituted of 2 syllables, had a late orthographic uniqueness point and few orthographic neighbors. The 50 pseudowords used for weak repetitions conditions were matched with word's lists for syllables and number of letters. For massively repeated items condition, one frequent word, one infrequent word and one pseudoword were selected. All were unused in weak repetition condition but were chosen to match to the characteristics of the lists. For the massively repeated condition with different fonts, three new items were chosen as previously. Twenty five different fonts were then applied to these three stimuli (figure 1). Fifty different fonts were initially used, but on the basis of pre-experimental behavioral data, the fonts that disrupted too much word recognition were removed and only 25 from them were finally selected.

### Procedure

The subjects were comfortably seated in the dark at a distance of 60 cm from a computer screen. The stimuli were white on a dark background with a visual angle subtending 2° and lasted 1 s, followed by a blank period lasting between 900 and 1100 ms during which the subjects stared at a fixation point. The subjects had to decide whether the stimulus was a word or not (lexical decision) by pressing one of two keys with their right hand.

The acquisition of behavioral and ERP data was separated by several weeks. Both behavioral and ERPs experiments were preceded by a training session.

### Behavioral study

For each participant, 20 frequent words, 20 infrequent words and 20 pseudowords were randomly selected among lists of 50 stimuli of the weak repetition condition. In the same way, 20 different fonts were selected among the 25 possibilities and were applied to the three stimuli for the repetition with different fonts condition. Stimuli of the massively repeated condition were repeated 20 times (see table 1). Presentation of all stimuli was mixed and no stimulus from a same condition was presented consecutively.

**Table 1:** Repetition conditions for behavioral and ERP study

	**Weak repetition**	**Massive repetition**	**Alternating fonts repetition**
**Behavioral study**	20 frequent words	1 frequent word × 20	1 frequent word × 20 different fonts
	20 infrequent words	1 infrequent word × 20	1 infrequent word × 20 different fonts
	20 pseudowords	1 pseudoword × 20	1 pseudoword × 20 different fonts
			
**ERP study**	50 frequent words × 2	1 frequent word × 100	1 frequent word × 25 different fonts × 4
	50 infrequent words × 2	1 infrequent word × 100	1 infrequent word × 25 different fonts × 4
	50 pseudowords × 2	1 pseudoword × 100	1 pseudoword × 25 different fonts × 4

A 3 modalities stimulus factor (frequent words/infrequent words/pseudowords) ANOVA was performed for each repetition condition on reaction times obtained for correct answers. A supplementary analysis was conducted in order to assess font alternation effects. This last analysis was applied only to massively repeated stimuli with and without alternating font repetitions with a 2 modalities font alternation factor (with/without) × 3 stimulus factor (frequent words/infrequent words/pseudowords) ANOVA.

### ERP study

During EEG acquisition, all 50 frequent words, 50 infrequent words and 50 pseudowords were presented twice in the weak repetition condition and repeated 100 times in the massive repetition condition. For the alternating font repetition condition, each font was presented 4 times (table 1).

**Figure 2:**
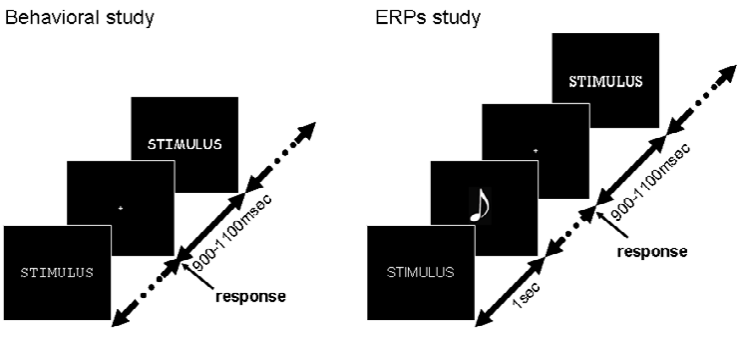
Design of behavioral and ERPs studies.

In order to avoid the recording of motor-related ERPs (see for example [32]), subjects responded to lexical decision only after hearing a beep sound announcing the end of word presentation during EEG acquisition (figure 2).

The EEG was recorded with 32 tin electrodes (electrocaps) from FP1, F7, F3, C3, T3, CP3, TP7, T5, PO5, PO3, P3, O1, XO1, FP2, F8, F4, C4, T4, CP4, TP8, T6, PO6, PO4, P4, O2, XO2, Fz, Cz, Cpz, Pz, Poz, and Oz sites distributed according to the 10–20 system. During acquisition, each electrode was referred to Cz. Electrode resistance was kept under 5 kΩ. The EEG was amplified, digitized at a rate of 256 Hz, filtered (band-pass 0.1 Hz – 100 Hz), and stored on the Deltamed™ software system.

The EEGs were averaged with a multi-electrode reference [34] composed of F7, F3, C3, T3, CP3, TP7, T5, P3, F8, F4, C4, T4, CP4, TP8, T6, P4, Fz, Cz, Cpz, and Pz sites. These electrode sites were chosen in order to obtain a uniform distribution on the scalp. Frequencies higher than 48 Hz were rejected. The baseline was calculated as the mean voltage during the 250 ms preceding the stimuli. Approximately 5% of the trials were excluded because of ocular artifacts, defined by amplitudes greater than 100 μV at FP1 and FP2 electrodes.

Because of the great differences between the repetition conditions, we performed statistical analyses separately for weak repetition condition, massive repetition, and massive repetition with font alternation. Moreover, we averaged EEG for the 50 first presentations and the 50 last presentations separately for these 3 conditions in order to have a better overview of the repetition effects.

Because of the goal of the study, we focused on the N170 component. Statistical analyses were performed on mean amplitudes collected between 140 – 280 ms for occipital (O1, XO1, O2, XO2) and posterior temporal electrodes (T5, PO5, T6, PO6) with a 2 average epoch factor (50 first stimuli/50 last stimuli) × 3 stimulus (frequent words/infrequent words/pseudowords) × 2 hemispheres (left/right) × 2 electrodes ANOVA.

When necessary, Tukey HSD post-hoc analyses were conducted.

## Results

### Behavioral data

**Figure 3:**
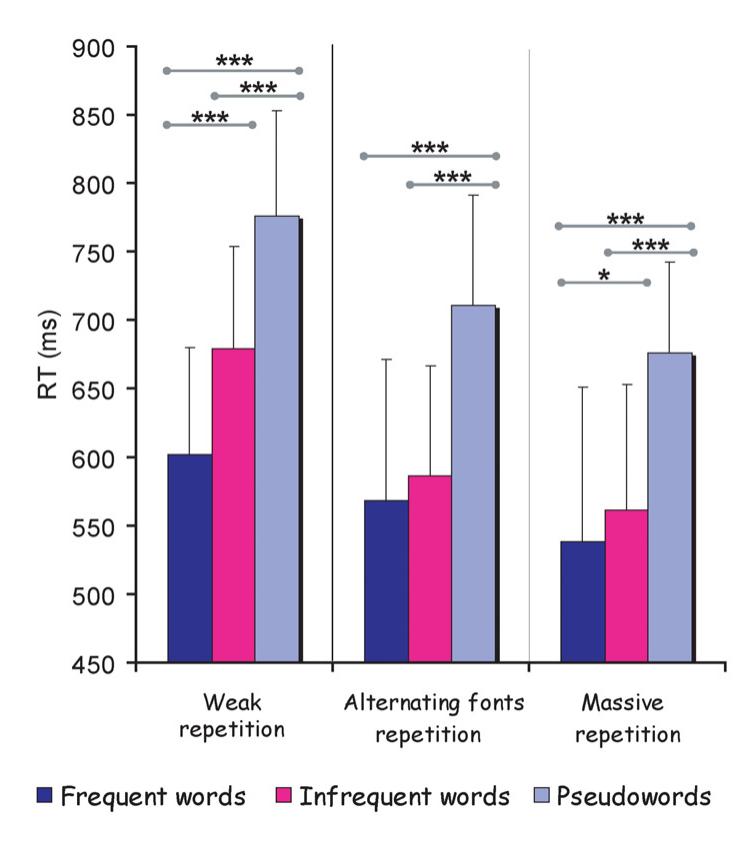
**Stimulus and repetition type effects on RTs**. Legends. ***: p < .001 ; *: p < .05

For the weak repetition, a stimulus main effect was found on RTs (F_2,54 _= 70.53, p < .001), as frequent words (m = 602 ms; SD = 78) elicited faster responses than infrequent ones (m = 679 ms; SD = 103) and pseudowords (m = 776 ms; SD = 133) (figure 3).

For massively repeated stimuli, a similar but attenuated pattern was obtained (F_2,54 _= 170.15, p < .001), responses to frequent (m = 538 ms; SD = 77) being faster than infrequent words (m = 562 ms; SD = 79.82) and pseudowords (m = 676 ms; SD = 66) (figure 3).

Concerning the alternating font repetitions, a stimulus main effect was also evidenced (F_2,54 _= 139.83, p < .001). However, post-hoc comparisons indicated that frequent word (568 ms ± 74) didn't differ significantly from infrequent ones (m = 586 ms; SD = 81) whereas these two types of words elicited faster responses than the pseudoword (m = 711 ms; SD = 92) (figure 3).

The comparison between massive repetition with and without alternating font evidenced a main effect of font alternation (F_1,27 _= 48.48, p < .001). Subjects responded faster after massive repetition (m = 592 ms; SD = 95) than alternating font condition (m = 622 ms; SD = 103). A stimulus effect (F_2,54 _= 282.65, p < .001) was also found as previously reported. The interaction repetition × stimulus was not significant.

### N170 electrophysiological data

Because of the great number of repetitions occurred during the ERP acquisition, the differences between the first 50 presentations and the last 50 were assessed in order to estimate the evolution of the repetition effect. In the weak repetition condition, the repetition effect consisted in the comparison of the first presentation of 50 stimuli contrasted to the second presentation of the same 50 stimuli. In massive repetition condition, the 50 first presentations of a unique stimulus were compared to the 50 last of the same unique stimulus. In alternating font repetitions condition, the 50 first presentations of a unique stimulus presented with different fonts were compared to the 50 last presentations of the same condition.

### Weak repetition

Analysis comparing different stimuli of the lists and the repetition (the difference between the first and the second presentation) of these stimuli evidenced a main effect of hemisphere at temporal (F_1,9 _= 5.41, p < .05) and occipital electrodes (F_1,9 _= 5.72, p < .05), as the negativity was larger on the left side.

**Figure 4:**
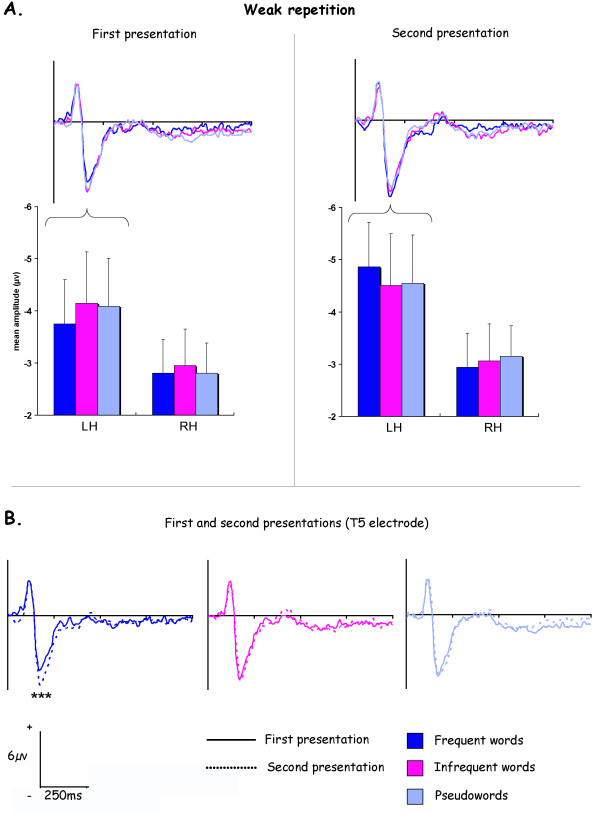
**ERPs results for the weak repetition**. A. Histograms represent N170 mean amplitudes averaged for T5-PO5 temporal electrodes at the left (LH) and T6-PO6 at the right hemisphere (RH). B. ERPs obtained for the first and the second presentation of the three lists of 50 stimuli. All ERPs illustrations corresponded to T5 electrode.

At temporal locations (figure 4A), an interaction between the stimulus type, the repetition and the hemisphere was evidenced (F_2,18 _= 5.48, p < .05). It corresponded to a larger negativity for the second presentation in comparison to the first one that was specific to frequent words (figure 4B) and observed only in left hemisphere (p < .001). Despite this strong repetition effect on frequent words, post-hoc analysis evidenced no significant word frequency effect on N170.

At occipital electrodes, an interaction was obtained between repetition and electrode (F_3,27 _= 3.00, p < .05), due to a larger negativity between the first and the second presentation of the stimuli that was greater for O1 and O2 locations than XO1 and XO2 ones.

### Massively repeated stimuli

Although words and pseudoword were repeated 100 times, an hemispheric main effect was also evidenced in temporal (F_1,9 _= 7.59, p < .05) and occipital (F_1,9 _= 7.28, p < .05) electrodes.

**Figure 5:**
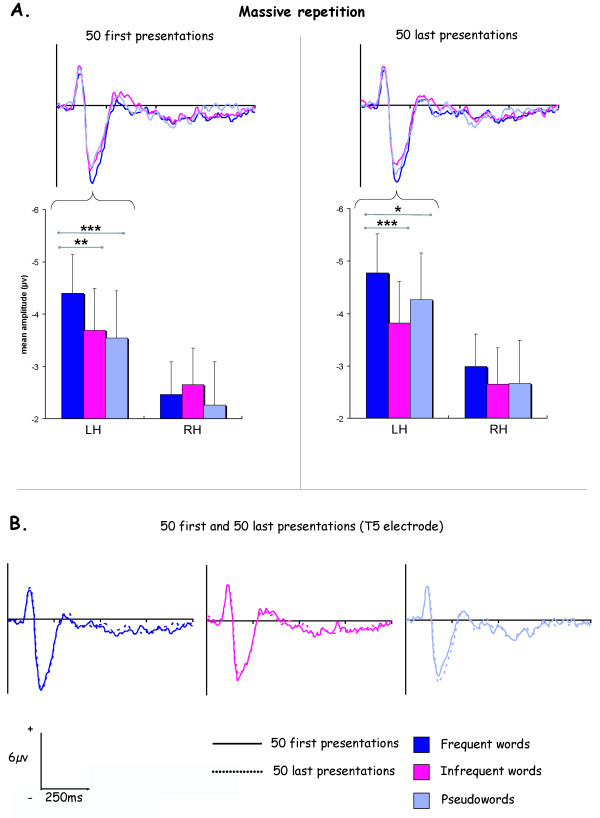
**ERPs results for the massively repeated stimuli**. A. Histograms represent N170 mean amplitudes averaged for T5-PO5 temporal electrodes at the left (LH) and T6-PO6 at the right hemisphere (RH). B. ERPs obtained for the 50 first and the 50 last presentations of the three massively repeated stimuli. All ERPs illustrations corresponded to T5 electrode.

At temporal location (figure 5), an interaction between stimulus and hemisphere (F_2,18 _= 4.69, p < .05) was obtained, frequent word eliciting larger negativity that infrequent word and pseudoword but only in the left hemisphere (p < .01). An interaction stimulus × repetition × hemisphere × electrode was also evidenced (F_2,18 _= 4.69, p < .05), word frequency effect being significant for all left temporal electrodes (T5 and PO5) during the 50 last presentations whereas this frequency effect was present only at T5 electrode for the 50 first presentations.

ERPs obtained at occipital electrodes were not modulated significantly by stimulus type or repetition.

### Alternating font repetitions

**Figure 6:**
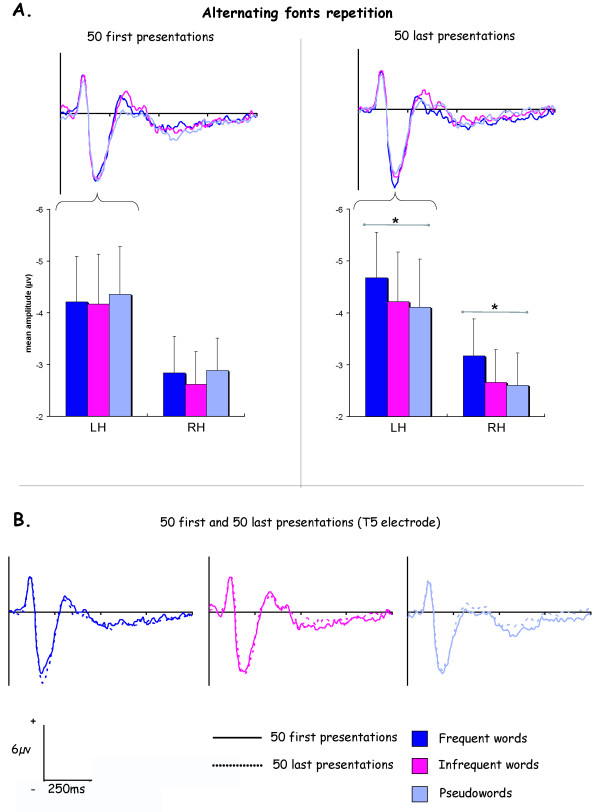
**ERPs results for the alternating fonts massive repetition**. A. Histograms represent N170 mean amplitudes averaged for T5-PO5 temporal electrodes at the left (LH) and T6-PO6 at the right hemisphere (RH). B. ERPs obtained for the 50 first and the 50 last presentations of the three stimuli repeated with different fonts. All ERPs illustrations corresponded to T5 electrode.

At temporal location, in addition to an hemispheric main effect (F_1,9 _= 5.67, p < .05), an interaction stimulus × repetition (F_2,18 _= 4.55, p < .05) was evidenced. Indeed, contrary to massive repetition condition, the N170 elicited during the 50 first repetitions with alternated fonts was not modulated by stimulus properties (figure 6). However during the 50 last repetitions, frequent words elicited larger negativity than pseudowords in left (p < .05) and right hemisphere (p < .05). Although frequent words generated larger amplitudes than infrequent ones, these differences didn't reach significance.

At occipital electrodes, only hemisphere (F_1,9 _= 9.93, p < .05) and electrode (F_3,27 _= 3.00, p < .05) main effects were significant.

## Discussion

### Behavioral data

We obtained a strong frequency effect in the case of weak repetition, frequent words eliciting faster RTs than infrequent ones (mean difference = 77 ms). This corresponds to the classical behavioral frequency effect obtained in lexical decision task well documented in literature [35-37]. Although the difference between frequent and infrequent words was not considerable in the massive repetition condition (mean difference = 24 ms), it remains significant. On the contrary, no frequency effect was evidenced when words were repeated with different fonts.

In fact, the comparison between massive repetition with and without font change demonstrated that the visual modifications of the word's shape minored repetition effects, with faster RTs for stimuli repeated without font modifications. Alterations of the shape of letters (changing fonts) slowed down the word recognition and attenuated the faster responses observed under a massive repetition, as described for case mixing [27,29]. These behavioral data are thus in agreement with the hypothesis that subjects adopt a visual discrimination strategy during identification of repetitive verbal stimuli.

This last assumption couldn't be assessed only on the basis of RTs examination. RTs represent a global view about the sum of the sensory and cognitive processes involved in the lexical decision task and the font alternation effect could occur at different stages of processing. ERPs results allowed us to study one temporal window about these stages.

### Electrophysiological data

Discrepancies exist between behavioral and ERPs data. However RTs represent the final step of a sum of process and frequency effect as well as repetition effect can occur at multiple levels. Because of the goal of this study our electrophysiological investigations focused only on an early component: the N170.

The results obtained for the weak repetition condition indicated that the N170 was not directly modulated by the word frequency. However, they evidenced a strong repetition effect specific to the frequent words, one repetition several minutes after the first presentation of the stimulus being enough to increase the negativity.

Most of fMRI works studying repetition effects reported a "suppression effect" consisting in a decrease of the signal in brain regions after the repetition of words [38] as well as faces [39]. This decrease of activity during the repetition was associated to a N400 repetition effect in electrophysiological studies [37,40-44]. On the contrary, our results demonstrated an early effect of the repetition that consisted in an increase of the amplitude for repeated stimuli. In fact these results are in agreement with the MEG study conducted by Dhond and colleagues [40] that evidenced that the earliest significant repetition effect during a paradigm of word stems generation appeared in the left posteroventral temporal cortex (centered in the lateral occipito-temporal sulcus) around 200 ms post-stimulus. As in the present study, they obtained a larger activation for repeated word stems in comparison to new word stems. According to Dhong and colleagues [45], when the repetition occurred 40 minutes after the stimulus presentation, then the repetition effect appeared only after 500 ms post-stimulus. Thus, the increase of the N170 amplitude during the repetition seems to be more linked to perceptive or lexicosemantic processes than long-term memory.

Under massive repetition condition, we observed a frequency effect for the N170, replicating results obtained in a previous study with different subjects and stimuli [11]. The modulation of the N170 amplitude by the word frequency under a massive repetition seems thus to be a robust effect. With 50 non-immediate repetitions, a word frequency effect appeared, a frequent word eliciting greater negativity than an infrequent word and a pseudoword. This effect was observed in the temporal electrodes and not in the occipital ones and was present only in the left hemisphere. In addition, although this word frequency effect occurs for the 50 first and the 50 last repetitions, it appeared more wide-spread at the temporal sites for the last ones, suggesting a cumulative effect.

These results could explain why some studies obtained a N170 word frequency effect while others did not. Indeed, MEG studies of Assadollahi and Pulvermuller [18,19] evidenced such a frequency effect at similar latencies of the N170 during a lexical decision using repeated stimuli. For the face, a similar N170 face familiarity effect was found during a massive repetition by Caharel and colleagues [20]. Therefore, the repetition seems to be a crucial element to observe a N170 word frequency effect and more generally a familiarity effect. This effect is probably also modulated by the task as revealed by a fMRI study of Henson and colleagues [39] with faces' stimuli.

The present results evidenced that some left temporal areas may produce more activity for words often encountered. However, this word repetition/frequency effect would not be consecutive to the VWFA activity alone. According to a fMRI study of Dehaene and colleagues [46], priming elicited reduction of activity in this area and this repetition effect was not modulated by letter case manipulation. These modulations of VWFA activity appear in contradiction with our results. We can hypothesize the existence of an early part of N170 component dedicated to a letter – visual pattern extraction whereas the late part corresponds to the VWFA involvement and to a more abstract representation of letters. These two processes would interact in a complex manner but the present word frequency/repetition effect would be related to the first stage of N170 component.

When alternating the font through a massive repetition, there was no difference between the frequent and infrequent words during the first 50 presentations in contrast to a "simple" massive repetition. This result suggests that the N170 frequency effect under a massive repetition has probably a perceptual source. This interpretation is strengthened by the study of Tanaka and Curran about object expertise because they support the view that enhanced N170 is probably the direct result of perceptual learning [47]. For the 50 last repetitions, we observed an increase of the N170 amplitude for the frequent word but not sufficient to evidence a significant frequency effect. However we obtained a difference between frequent words and pseudowords. We can thus assume that if we choose a greater number of stimulus exposures, the word frequency effect would be significant.

In fact our data can be interpreted according to several theoretical frameworks that we address thereafter.

### The magnocellular vs parvocellular hypothesis

Whereas numerous models of visual word recognition postulate the existence of two pathways in reading such as a lexical route and a second one applying grapheme-phoneme conversion rules in order to explain word frequency effect [48,49], our data suggest a more visual-basis effect. This effect may be explained by a parvo/magnocellular modulation in the visual word recognition. For example, according to Allen and colleagues [50], a word can be visually recognized either from its letter encoding or from its specific spatial frequency. The "letter-by-letter" route is slow, corresponding to the parvocellular pathway, as opposed to the faster holistic magnocellular system. In this model, familiar words may be identified by a global mode and infrequent words by a letter-by-letter analytic mode. A differential involvement of magno- and parvocellular pathway can probably modulate the N170, because a study of Torriente and colleagues [51] showed greater N170 amplitudes when subjects detected movements relative to colors of bars.

Moreover although very conflictual, some data suggested a link between magnocellular system impairment and developpemental dyslexia [52]. According to Vidyasagar [53], the interaction of the parvo and magnocellular system would play a crucial role in the early visual analysis of words and reading. In the light of these results, the larger amplitudes for frequent than infrequent words in the present study may correspond to the greater involvement of the magnocellular pathway at a logographic/holistic stage.

The absence of difference between the frequent and infrequent words during the first 50 repetitions with fonts mixing is also in agreement with this interpretation because the letter shape modification is more able to disrupt the fast magno- than parvocellular pathway. But how can we explain that this frequency effect appeared only during massive repetition? We can assume that these results are due to a threshold effect. Indeed, it is possible that very frequent words such as articles or prepositions generate holistic processing without a repetition, but because of the weak number of those stimuli and their great heterogeneity, ERPs technique doesn't allow us to test such a hypothesis.

### Global vs local processing

Electrophysiological literature contrasting both local (e.g. letter level) and global (e.g. whole word form) processing evidenced conflictual results. Some studies have found larger amplitudes during local than global processing for the P100 but not for the N170 component [54,55]. Some others have found such effects at longer latencies [56,57] or on the N170 but in an opposite manner to ours, i.e. an enhanced negativity for the local processing [58,59]. These discrepancies among those previous results but also with the present data may be explained by the stimuli and the tasks used. In these studies, visual objects composed of local elements that are spatially arranged to form a global shape were presented to subjects who had to pronounce on either local or global attributes. Such a global/local task is very different from the lexical decision that we used, because in the former one's attention is explicitly directed to local or global attributes (goal-directed attention) whereas in the later global/local processing would be function of stimulus (stimulus-driven attention). Moreover, a study of Evans and colleagues [60] can help us to understand why the N170 component was not always modulated by the global/local selective attention. They found greater N170 amplitude for global attention than for the local one but only when distractor elements (e.g. local elements during a global task) were invariant in the block of stimuli, in other words when distractor elements were repeated. Thus, it is possible that the enhanced negativity for frequent words would be due to specific global/holistic processing. According to the preceding authors, this greater amplitude could be interpreted in terms of a larger attentional window in the global processing than in the local one.

Usually, the global/local processing are associated to the right/left hemispheres, respectively [60-62]. However, Fink and colleagues [63] observed an effect of stimulus category on hemispheric specialization for global and local processing. Because of the well-known left hemisphere specialization and advantage in word processing, this leftward asymmetric effect seems consistent with our global interpretation. Indeed the hemisphere that is specialized in the word recognition is the more likely to engage fast global/holistic processing. It is also in agreement with the hemifield presentation study of Lavidor and colleagues [64] which suggested that the left hemisphere has a greater sensitivity to words presented in a familiar format. The development of such a sensitivity is due to the continuous exposure to written words and at the end only the left hemisphere recognizes words by direct addressing to lexicon (see also [28]).

### Expertise effects

Because some left occipito-temporal areas may be specialized in visual word recognition and orthographic skills [65], the repetition of invariant perceptive elements may improve the level of expertise of the subjects or increase the level of familiarity and thereby generating greater activities at this location. One study reported a significant interaction on the N170 amplitude evoked by birds and dogs and the level of expertise of a group of participants that included bird watchers and dog breeders [47]. When the subjects attained a high level of expertise, the N170 amplitude was larger, suggestive of a superior perceptual learning. Likewise, the level of expertise may change the manner in which the visual stimuli are processed [66]. It could then be assumed that the larger negativity during a massive repetition would be due to a "perceptual learning".

Recently two ERPs studies compared letter perception/reading in two languages using different alphabets in mono and bilingual subjects [67,68]. They evidenced a larger N170 amplitude in monolingual subjects reading letterstrings in their native language in comparison to letterstrings of a foreign language using different alphabet such as Chinese or Arabic for English or French subjects respectively. On the contrary, such differences were absent in bilingual subjects. These results are in agreement with the expertise hypothesis and suggest that the subjects develop skilled process, especially in the left hemisphere, to fast recognize letters of a language for witch they have a great exposure. Our protocol contained massively repeated stimuli that permitted to simulate at a lesser extend this continuous exposure to written words during reading acquisition. As a matter of fact our results evidenced that this repetition enhances the N170 amplitude of frequent words as the expertise do. However one of the major questions that further studies would have to answer is to know if this expertise effect depends exclusively on the repetition or if the knowledge about stimuli is crucial to obtain this effect.

## Conclusion

The N170 component represents a major step in the visual word recognition. The effects of the repetition, the word frequency and the word visual shape manipulation evidenced on this component suggest the existence of more than one unique way to encode a string of letters. Further studies should investigate the impact of the age of word acquisition on N170 because we can hypothesize that the holistic logographic process could depend on a critical period during the childhood. Recently, Fiebach and colleagues [69] attempted to isolate the brain areas more activated for early learned words in comparison to later ones during a visual lexical decision task and suggested that reading an early learned word actives the sound structure of this word or the semantic knowledge in a more direct way. All these data converge to the interpretation that the early acquisition of a word, its great expertise, and its high frequency favor the use of a more global/holistic processing of words and the present N170 amplitude increase reflects such a processing.

## Competing interests

The author(s) declare that they have no competing interests.

## Authors' contributions

GS elaborated stimuli, participated to EEG acquisition and post-processing, statistical analysis, redaction of the manuscript and created figures. LP participated to the redaction of the manuscript. CB created software for stimuli presentation and ERP processing. MR participated in the design of the study.
